# Stability of azasetron-dexamethasone mixture for chemotherapy-induced nausea and vomiting administration

**DOI:** 10.18632/oncotarget.22174

**Published:** 2017-10-31

**Authors:** Bao-Xia Fang, Fu-Chao Chen, Dan Zhu, Jun Guo, Lin-Hai Wang

**Affiliations:** ^1^ Department of Pharmacy, Dongfeng Hospital, Hubei University of Medicine, Shiyan, Hubei 442008, P.R. China; ^2^ Department of Pharmacy, Renmin Hospital, Hubei University of Medicine, Shiyan, Hubei 442000, P.R. China; ^3^ Department of Oncology, Dongfeng Hospital, Hubei University of Medicine, Shiyan, Hubei 442008, P.R. China

**Keywords:** chemotherapy-induced nausea and vomiting, antiemetic, dexamethasone, azasetron, drug stability

## Abstract

Combination antiemetic therapy has become common practice for the prevention of nausea and vomiting caused by anticancer drugs. In this study, we investigated the stability of azasetron hydrochloride 0.1 mg/mL plus dexamethasone sodium phosphate 0.05, 0.1, or 0.2 mg/mL in 0.9% sodium chloride injection and stored in polyolefin bags and glass bottles over a period of 14 days at 4°C and 48 hours at 25°C. The stability studies were evaluated by visual inspection, pH measurement, and a high-pressure liquid chromatography assay of drug concentrations. During the study period, the concentration of each drug in the various solutions remained above 97% of the initial concentration at both 4°C and 25°C when protected from room light. Under the condition of 25°C with exposure to room light, the concentrations of both drugs were significantly lowered over 48 hours. The pH value decreased, and the color changed from colorless to pink. Our study demonstrates that the azasetron-dexamethasone mixture at a clinically relevant concentration seems to be stable for 48 hours at 25°C and for 14 days at 4°C when packaged in polyolefin bags or glass bottles and protected from room light. The room light is the main influential factor on stability. Clinicians should be aware that combinations of azasetron hydrochloride and dexamethasone sodium phosphate in solution with light exposure should be avoided.

## INTRODUCTION

Chemotherapy-induced nausea and vomiting (CINV) are common side effects of many antineoplastic regimens in the treatment of cancer and can last for days following therapy, result in various metabolic and nutritional status, and sometimes even discontinue or postpone patients’ chemotherapy [1–3]. Currently, adherence to antiemetic guidelines provides effective relief from CINV, and patients rapidly return to normal daily activities after treatment [4–6]. According to antiemetic guidelines, combination antiemetic therapy has become common practice for the prevention of CINV, and the effectiveness of the type 3 serotonin receptor antagonists (5-HT_3_ RA) has been enhanced by combination with dexamethasone [1, 7–9].

Azasetron, a selective potent 5-HT_3_ RA, is a derivative of benzamide with a different chemical structure from other 5-HT_3_ RA, such as ondansetron, granisetron, tropisetron, and ramosetron, and has a longer duration of action and a higher affinity for the 5-HT_3_ receptor [10]. Azasetron was first approved in 1994 by the Yoshitomi Pharm, Ind., Ltd. and Japan Tobacco Inc and is gaining popularity in Asia Pacific countries as an antiemetic for the prevention of CINV and postoperative nausea and vomiting [11–15]. Dexamethasone, a synthetic corticosteroid, has long been used as an antiemetic agent in patients undergoing cancer chemotherapy [16, 17], being effective for both acute and delayed nausea and vomiting. A survey of the literature revealed that co-administration of azasetron with dexamethasone was more effective in reducing CINV compared to azasetron alone in patients undergoing cisplatin chemotherapy [14, 15]. Currently, there are no commercially available antiemetic combination mixtures, and they must be prepared in the hospital pharmacy intravenous admixture service for clinical use. Known incompatibilities, such as color change, pH shifts, precipitation, particle formation, gas evolution, and crystallization during i.v. administration arising from the combination of therapies can exist [18]. Hence, knowledge of the compatibility and stability of this drug combination is important.

From the literature survey, several studies have assessed the stability and compatibility of dexamethasone combines with 5-HT_3_ RA, such as ondansetron, granisetron, tropisetron, or palonosetron hydrochloride in binary admixtures [19–27]. The stability of azasetron with dexamethasone in solution for CINV administration has not been reported. Thus, the aim of this study was to determine the stability of the two drugs, at 3 different concentration combinations, prepared in 0.9% sodium chloride injection and stored in 2 different types of containers (glass bottles and polyolefin bags) for a period of 14 days at 4°C protected from room light and 48 hours at 25°C protected from room light or exposure to room light.

## RESULTS

### Physical compatibility

**Figure 1:**
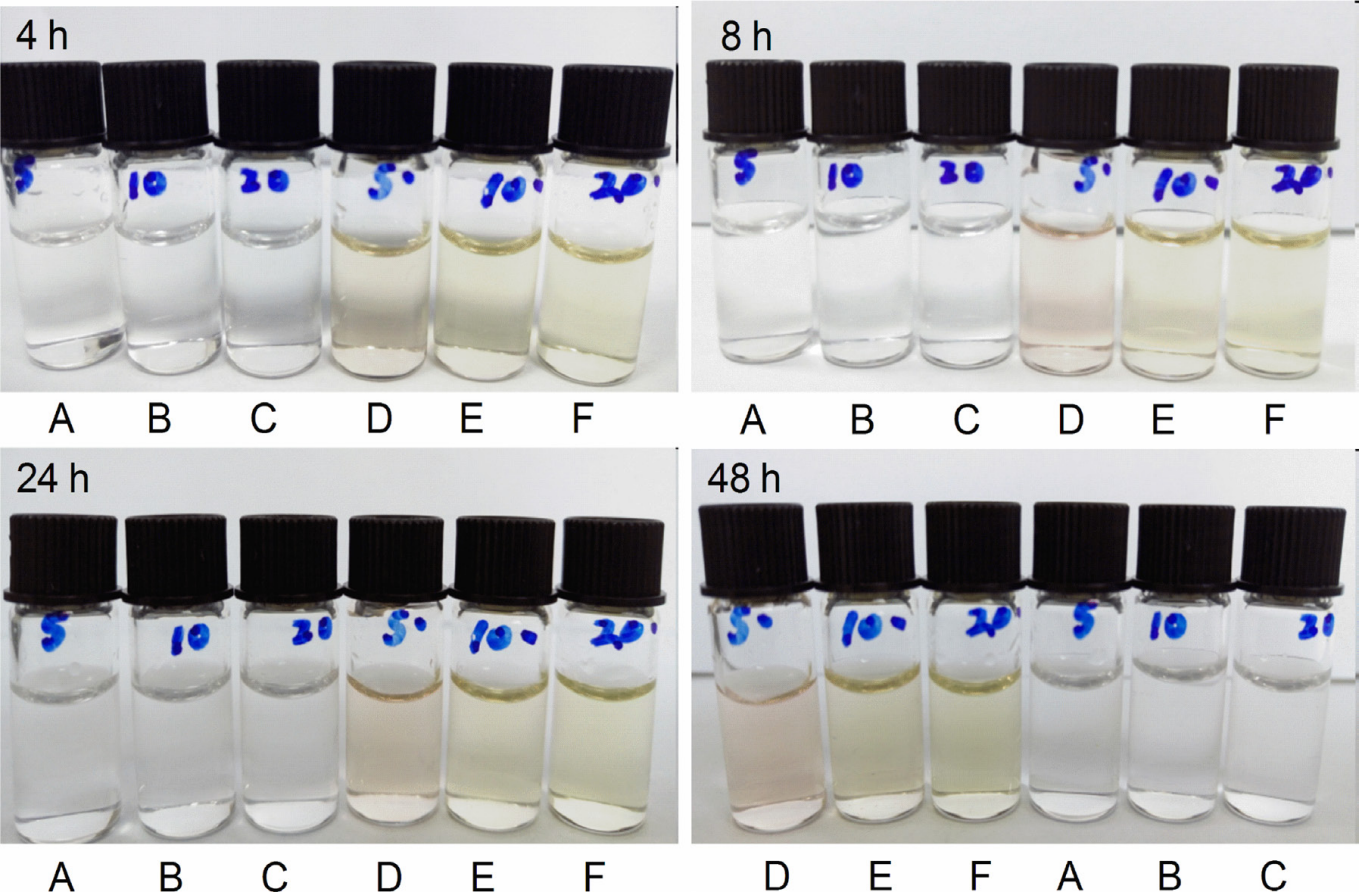
The color changes of the azasetron-dexamethasone mixtures at 4 hr, 8 hr, 24 hr and 48 hr after preparation (**A**) AD5 (25°C, protected from room light); (**B**) AD10 (25°C, protected from light); (**C**) AD20 (25°C, protected from room light); (**D**) AD5 (25°C, exposed to room light); (**E**) AD10 (25°C, exposed to room light); (**F**) AD20 (25°C, exposed to room light). AD5: azasetron hydrochloride 0.1 mg/mL plus dexamethasone sodium phosphate 0.05 mg/mL in 0.9% sodium chloride injection; AD10: azasetron hydrochloride 0.1 mg/mL plus dexamethasone sodium phosphate 0.1 mg/mL in 0.9% sodium chloride injection; AD20: azasetron hydrochloride 0.1 mg/mL plus dexamethasone sodium phosphate 0.2 mg/mL in 0.9% sodium chloride injection.

During the stability study period, no precipitate was visible in any solution, no color changes occurred, no gas was produced, and the pH values remained stable at both 4°C and 25°C when protected from room light. In contrast, when stored at 25°C and exposed to room light, the pH value of the infusion mixtures decreased, and the color changed from colorless to pink. The results of the pH value for azasetron-dexamethasone mixtures in polyolefin bags and glass bottles at 25°C and exposed to room light are presented in Table 1. As indicated in Table 1, all drug mixtures were nearly neutral with pH values ranging from 6.8–7.2 at the start of the study as a consequence of increasing dexamethasone sodium phosphate concentrations in the mixtures. The pH changes were 0.9–1.2 units of the initial pH for all drug mixtures at 25°C with exposure to room light. The color changes of the infusion mixtures at 25°C and exposed to room light are illustrated in Figure 1. From Figure 1 we can see that all the mixtures were clear, colorless at the beginning. After 4 hours, the presence of a canary yellow was observed. At the end of the study (48 hours), the color changed from yellow to pink when stored at 25°C with light exposure.

**Table 1:** pH values of azasetron-dexamethasone mixtures based on three different concentrations packaged in polyolefin bags or glass bottles and exposed to room light at 25°C over 48 hours

Mixture and Container	pH value (mean ± SD; *n* = 3)				
	0 hours	4 hours	8 hours	24 hours	48 hours
*Glass bottles*					
AD5	6.89 ± 0.08	6.32 ± 0.06	6.16 ± 0.04	6.03 ± 0.05	5.84 ± 0.04
AD10	7.00 ± 0.04	6.44 ± 0.09	6.11 ± 0.05	5.95 ± 0.03	5.82 ± 0.09
AD20	7.24 ± 0.06	6.65 ± 0.04	6.38 ± 0.07	6.14 ± 0.04	6.08 ± 0.06
*Polyolefin bags*					
AD5	6.80 ± 0.06	6.47 ± 0.09	6.13 ± 0.05	6.02 ± 0.03	5.90 ± 0.04
AD10	7.03 ± 0.07	6.55 ± 0.08	6.27 ± 0.04	6.10 ± 0.05	5.98 ± 0.02
AD20	7.19 ± 0.04	6.81 ± 0.05	6.56 ± 0.05	6.35 ± 0.06	6.14 ± 0.03

### Chemical stability study of azasetron and dexamethasone at 4°C

The results of the chemical stability study for the admixtures of azasetron and dexamethasone in polyolefin bags and glass bottles when stored at 4°C over 14 days and protected from room light are summarized in Table 2. As shown in Table 2, losses of dexamethasone sodium phosphate and azasetron hydrochloride were less than 3% in 3 different concentration combinations stored in polyolefin bags and glass bottles over a period of 14 days at 4°C and protected from room light.

**Table 2:** Stability of azasetron-dexamethasone mixtures based on three different concentrations packaged in polyolefin bags or glass bottles, and protected from room light over 14 days at 4°C

Mixture and Container	Drug	Initial Concentration Remaining (Mean ± S.D. %; *n* = 3)				
		Day 1	Day 3	Day 7	Day 10	Day 14
*Glass bottles*						
AD5	azasetron	100.63 ± 0.2	99.93 ± 0.1	99.35 ± 0.3	99.78 ± 0.2	98.19 ± 0.4
	dexamethasone	100.84 ± 0.3	100.39 ± 0.2	100.73 ± 0.4	99.04 ± 0.5	99.62 ± 0.3
AD10	azasetron	100.53 ± 0.3	100.55 ± 0.2	100.84 ± 0.4	100.74 ± 0.4	99.95 ± 0.2
	dexamethasone	99.28 ± 0.5	99.37 ± 0.2	99.81 ± 0.6	100.73 ± 0.4	100.91 ± 0.2
AD20	azasetron	100.63 ± 0.2	100.94 ± 0.2	100.73 ± 0.3	100.85 ± 0.5	98.05 ± 0.6
	dexamethasone	99.36 ± 0.1	99.80 ± 0.2	99.05 ± 0.2	99.15 ± 0.2	99.63 ± 0.3
*Polyolefin bags*						
AD5	azasetron	100.96 ± 0.2	101.04 ± 0.1	101.47 ± 0.3	100.96 ± 0.2	99.54 ± 0.2
	dexamethasone	99.71 ± 0.1	99.92 ± 0.2	99.99 ± 0.2	100.06 ± 0.4	99.53 ± 0.2
AD10	azasetron	100.90 ± 0.4	100.69 ± 0.2	100.41 ± 0.2	100.44 ± 0.5	100.67 ± 0.2
	dexamethasone	99.66 ± 0.5	99.58 ± 0.2	99.49 ± 0.3	99.60 ± 0.3	99.60 ± 0.5
AD20	azasetron	100.89 ± 0.2	100.27 ± 0.2	100.2 ± 0.1	100.04 ± 0.4	97.85 ± 0.2
	dexamethasone	99.87 ± 0.1	99.90 ± 0.3	99.76 ± 0.2	99.99 ± 0.2	99.19 ± 0.5

### Chemical stability study of azasetron and dexamethasone at 25°C

**Figure 2:**
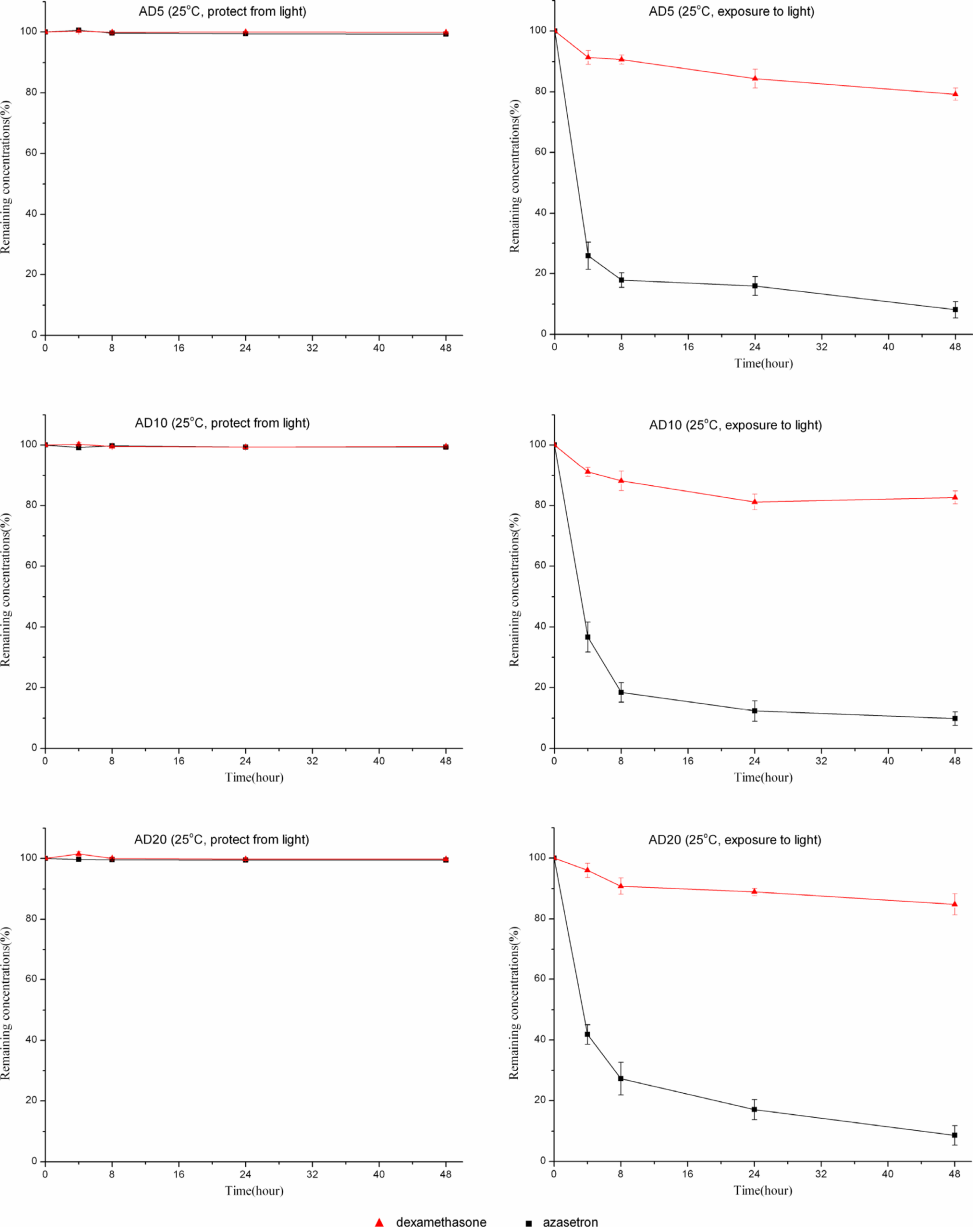
The stability of azasetron-dexamethasone mixtures based on three different concentrations packaged in glass bottles and stored at 25°C, under light exposure or light protection conditions, for 48 hours Error bars represent the standard deviation (*n* = 3).

**Figure 3:**
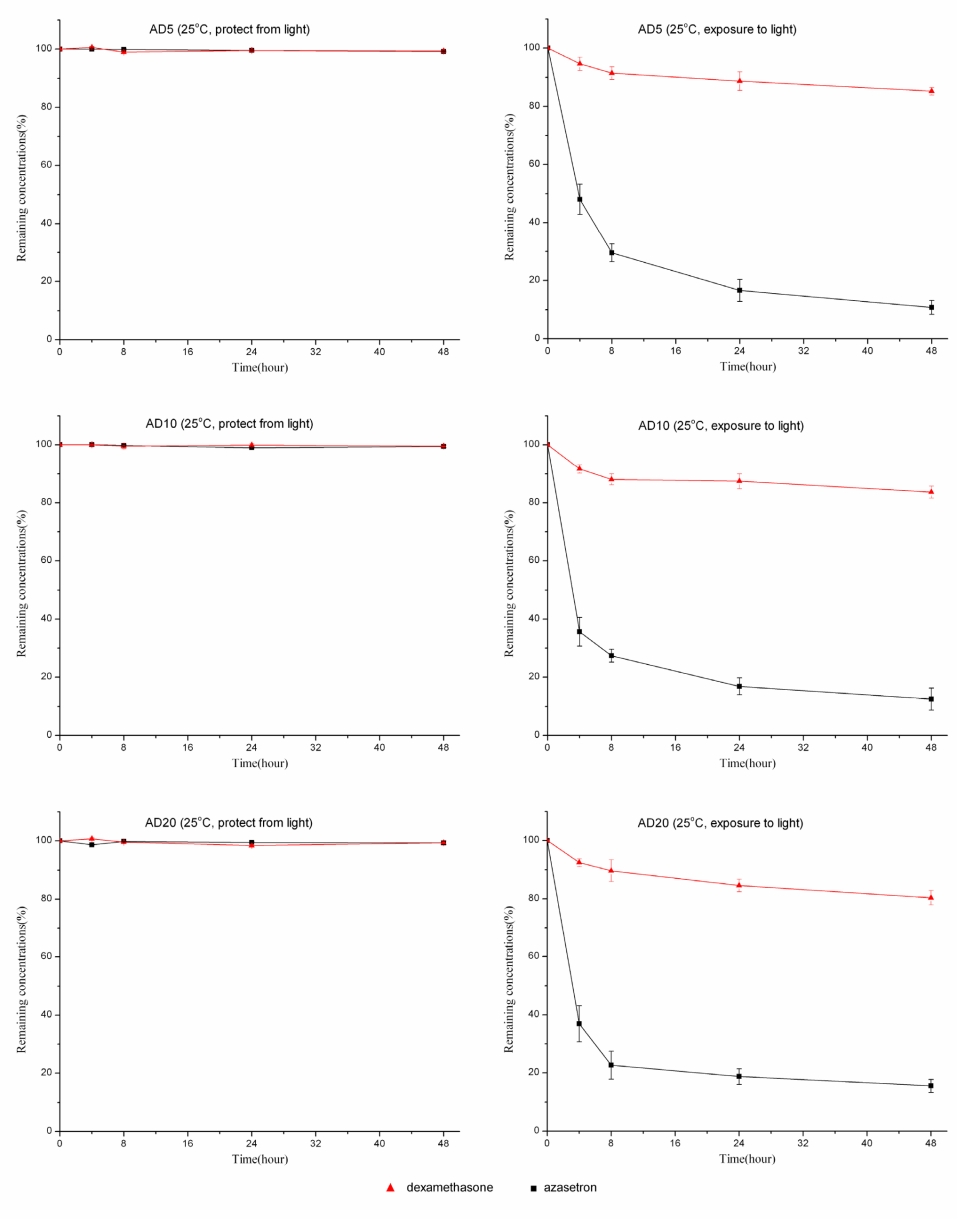
The stability of azasetron-dexamethasone mixtures based on three different concentrations packaged in polyolefin bags and stored at 25°C, under light exposure or light protection conditions, for 48 hours Error bars represent the standard deviation (*n* = 3).

At room temperature, solutions of dexamethasone sodium phosphate (0.05, 0.1, or 0.2 mg/mL) combined with azasetron hydrochloride (0.1 mg/mL) were also stable for 48 hours when packaged in polyolefin bags or glass bottles and protected from room light (Figures 2, 3). Under the condition of light exposure, the concentrations of both drugs were significantly lowered over 48 hours. Figures 2, 3 show the variation in the remaining concentrations of azasetron hydrochloride and dexamethasone sodium phosphate in polyolefin bags or glass bottles that were exposed to room light during the time of the study; as can be observed, remaining concentrations of azasetron hydrochloride were lower than 50% after 4 hours of storage. After 24 hours, losses of azasetron hydrochloride and dexamethasone sodium phosphate were more than 70% and 10%, respectively. At the end of the study (48 hours), losses of azasetron hydrochloride and dexamethasone sodium phosphate were more than 80% and 20% in both polyolefin bags and glass bottles.

## DISCUSSION

CINV is one of the most debilitating adverse events of chemotherapy in cancer patients. Several neurotransmitters, including dopamine, serotonin, and substance P, have been identified as important mediators of CINV [1, 2]. Hence, many treatment guidelines recommend that co-administration of antiemetics from different classes could be a more effective antiemetic treatment modality, and dexamethasone is a standard component of antiemetic combination regimens with 5-HT_3_ antagonists [6–8, 28, 29]. Fujii M *et al* [14] found that azasetron combined with dexamethasone was more effective than azasetron alone for prevention of cisplatin-induced emesis in patients with advanced head and neck carcinoma. Lee *et al* have administered azasetron combined with dexamethasone and combinations of ondansetron and dexamethasone in the prevention of delayed CINV and have found that azasetron showed inferiority in the control of delayed CINV compared with ondansetron [15].

In everyday clinical practice, co-administration of antiemetic drugs in the same intravenous infusion solution is often highly preferred to improve the management of ambulatory procedures, time management and the number of administered intravenous preparations [22]. All this would improve patient safety and comfort. To our knowledge, no published information is available on the compatibility and stability of dexamethasone in combination with azasetron in infusion solution. The aim of this study was to address this lack of information.

To date, there is limited published information concerning the compatibility and stability of azasetron hydrochloride, either singly or combined with other drugs in infusion solutions. Wang DW *et al* [30] studied the effect of light and temperature on the stability of azasetron hydrochloride prepared in 0.9% sodium chloride solutions or 5% glucose injection. They reported that azasetron hydrochloride was stable for 24 h when stored at 4°C, 25°C or 35°C with protection from room light. The concentration of azasetron was significantly lost in normal saline and glucose injection 4 h after mixture, and the color changed from colorless to pink when stored at 25°C or 35°C with light exposure. In the present study, all azasetron-dexamethasone admixtures showed similar results in normal saline with light exposure. Our study identified that room light is an important factor that affects the mixture’s stability, and therefore, all dexamethasone-azasetron admixtures prepared for clinical use should be protected from room light to avoid this destabilizing factor.

Regarding dexamethasone, the stability and compatibility of dexamethasone sodium phosphate, either singly or combined with other drugs in infusion solutions, have been widely tested and studied. Dexamethasone sodium phosphate degradation was less than 3% when diluted in 0.9% sodium chloride injection and stored in polypropylene syringes for 22 days at 25°C [31]. The combination of dexamethasone sodium phosphate with certain drugs in infusion solutions has displayed variable results. It has been proven to be stable with ketamine hydrochloride, metoclopramide hydrochloride, granisetron hydrochloride, ondansetron hydrochloride, palonosetron hydrochloride, tramadol hydrochloride, diphenhydramine hydrochloride, lorazepam, and cefpirome sulfate, among others [19–26, 32–37], whereas it precipitates at certain concentrations when combined in solution with morphine hydrochloride, hydromorphone hydrochloride, midazolam hydrochloride, haloperidol lactate, propofol, or fenoldopam mesylate [38–43].

In this stability study, we have examined only physicochemical stability without considering microbial contamination. In clinical practice, we should follow the United States Pharmacopeia and National Formulary (USP/NF) Chapter 797 [44]. Under this regulation, the preparation is categorized as a low-risk compounding sterile product [45]. To ensure its safety, the preparation should be used by 48 hours beyond the date at room temperature or by 14 days beyond the date at refrigerated temperatures, based on USP specifications. The results of our stability studies show that infusion solutions containing concentrations of azasetron hydrochloride 0.1 mg/ml combined with dexamethasone sodium phosphate (0.05–0.2 mg/ml) were very stable for 48 hours at 25°C and for 14 days at 4°C when packaged in polyolefin bags or glass bottles and protected from room light. Clinicians should be aware that there may be significant loss of azasetron and dexamethasone with light exposure. Based on this finding, it is recommended that, whenever possible, combinations of azasetron and dexamethasone in solution with light exposure should be avoided.

## MATERIALS AND METHODS

### Chemicals and reagents

The working standards of dexamethasone sodium phosphate (99.8%, HPLC-grade) and azasetron hydrochloride (99.7%, HPLC-grade) were purchased from the National Institutes for Food and Drug Control (Beijing, China). The pharmaceutical formulations used in this study were dexamethasone sodium phosphate injection 5 mg/1 mL (Cisen Pharmaceutical Co., Ltd., Shandong, China) and azasetron hydrochloride injection 10 mg/2 mL (Zhejiang Wanma pharmaceutical Co., Ltd., Hangzhou, China). The solution of 0.9% sodium chloride injection used to prepare the sample mixtures was from Kelun Pharmaceutical Co., Ltd. (Sichuang, China).

### Analytical method

The concentrations of azasetron hydrochloride and dexamethasone sodium phosphate were determined using a previously described high performance liquid chromatography (HPLC) method [27]. Chromatography equipment included a Shimadzu LC-20A (Shimadzu, Kyoto, Japan) LC system composed of a LC-20AD quaternary pump, a DGU-20A5 degasser unit, a SIL-20AC auto-injector, a CTO-20A column oven and a SPD-M20A diode array detector (DAD). Data acquisition was carried out using Class VP 7.4 software (Shimadzu, Kyoto, Japan). Dexamethasone sodium phosphate was separated from azasetron hydrochloride by HPLC on a phenomenex C_18_ column (4.6 mm × 150 mm, 5 μm) with mobile phase of acetonitrile, 50 mM KH_2_PO_4_ buffer, and triethylamine (25: 74: 1; v/v). The pH was adjusted to 4.0 with 85% phosphoric acid. Flow rate was 1.0 mL/min, and the detection wavelengths for dexamethasone and azasetron were 241 nm and 302 nm, respectively. The assay was performed at 30°C and the injection volume was 20 μL. The degraded solution of azasetron hydrochloride or dexamethasone sodium phosphate was used to prove the specificity of the HPLC method. This solution was incubated in1N hydrochloric acid, 1N sodium hydroxide, 3% hydrogen peroxide for 5 hours at 60°C. The chromatogram of each admixture showed retention times of 4.5 min and 10.7 min for azasetron and dexamethasone, respectively. Degradation peaks were detected which did not interfere with the corresponding drug.

### Preparation of dexamethasone-azasetron solutions

To mimic a concentration range relevant to clinical practice and the conditions commonly occurring in hospitals [14–17], 3 different concentration combinations were freshly prepared under aseptic conditions in laminar flow hoods, kept in the dark under refrigeration (4 ± 0.5°C) for 14 days and protected from room light or exposed to room light at room temperature (25 ± 0.5°C) for 48 hours.

Solution 1 (AD5): 1 mL (5 mg) dexamethasone sodium phosphate injectable solution and 2 mL (10 mg) azasetron hydrochloride injectable solution were transferred to 100 mL polyolefin bags or glass bottles and filled with 0.9% sodium chloride injection.

Solution 2 (AD10): 2 mL (10 mg) dexamethasone sodium phosphate injectable solution and 2 mL (10 mg) azasetron hydrochloride injectable solution were transferred to 100 mL polyolefin bags or glass bottles and filled with 0.9% sodium chloride injection.

Solution 3 (AD20): 4 mL (20 mg) dexamethasone sodium phosphate injectable solution and 2 mL (10 mg) azasetron hydrochloride injectable solution were transferred to 100 mL polyolefin bags or glass bottles and filled with 0.9% sodium chloride injection.

### Stability of dexamethasone and azasetron mixture

Following the American Society of Health-System Pharmacists guidelines [45, 46], stability studies were conducted in triplicate for each type of storage condition and container. The physical characteristics of the solutions, such as color, cloudiness, and precipitation were evaluated qualitatively at the time of preparation and at the specified times. Visual examinations were assessed in normal diffuse fluorescent room light with the unaided eye against white and black backgrounds. The pH values of the mixture were also determined by a PHS-3c pH meter (Leici Instrument Co., Shanghai, China). Furthermore, the concentrations of the drugs were determined at each analysis by the above described HPLC–DAD method. In the concentration analyses, 2 mL samples were collected from each polyolefin bag or glass bottle and were allowed to reach room temperature before injection into an HPLC system. Samples from each syringe were analyzed in triplicate (total *n* = 3). The initial concentrations of dexamethasone sodium phosphate and azasetron hydrochloride were defined as 100%, and subsequent sample concentrations for every drug in the mixtures were reported as the percentage of the initial concentration. Stability was defined as the retention of at least 90% of the initial drug concentration.
